# Strain analysis of cardiac chambers in individuals with temporal lobe epilepsy

**DOI:** 10.1111/epi.18472

**Published:** 2025-06-13

**Authors:** Ramsés Miotto, Márcia Tatsch Cavagnollo, Katia Lin, Guilherme L. Fialho

**Affiliations:** ^1^ Cardiology Division Federal University of Santa Catarina Florianopolis Brazil; ^2^ Neurology Division Federal University of Santa Catarina Florianopolis Brazil; ^3^ Center for Applied Neurosciences Federal University of Santa Catarina Florianopolis Brazil; ^4^ Medical Sciences Postgraduate Program Federal University of Santa Catarina Florianopolis Brazil

**Keywords:** arrhythmia, epilepsy, heart disease, heart failure, myocardial strain

## Abstract

Cardiovascular impairment in people with epilepsy (PWE) has been attracting medical interest. This study investigated the myocardial strain of the four cardiac chambers of individuals with temporal lobe epilepsy, without known heart disease, compared to an age‐, sex‐, and body mass index‐matched control group. Eighty‐six individuals (43 with epilepsy and 43 controls) underwent clinical evaluation, electrocardiography, and transthoracic echocardiography. Individuals with epilepsy had reduced left ventricular ejection fraction (*p* = .001), left ventricular longitudinal strain (*p* = .011), right ventricular strain (*p* = .024), left atrial biplanar contractile strain (*p* = .009), and right atrial reservoir (*p* = .0001), conduit (*p* = .025), and contractile strain (*p* = .0003). In multivariate analysis, epilepsy was a predictor of reduced contractile and reservoir strain values of both atria. Among individuals with epilepsy, polytherapy with antiseizure medications was an independent predictor of reduced left ventricular global longitudinal strain. Disease duration was an independent predictor of reduced left and right atrial reservoir strain and right atrial contractile strain. These data suggest subtle and early cardiac involvement in PWE and could help clarify the increased incidence of cardiac events in this population.

## 1 INTRODUCTION

Epilepsy affects approximately 50 million people worldwide, being one of the most significant neurological conditions in terms of disability‐adjusted life‐years lost.^1^ Furthermore, people with epilepsy (PWE) have a 1.6–3.0 increased risk of premature death from all causes, and a 3 times greater risk of sudden cardiac death compared to people without epilepsy.^1^, ^2^ Recurrent epileptic seizures leading to an autonomic surge, associated with catecholaminergic toxicity and hypoxemia, may cause small and cumulative damage to the heart and coronary vessels, causing electromechanical dysfunction. This concept was designated “epileptic heart.”^3^

Epilepsy‐associated cardiac damage evaluated through myocardial strain is not fully explored. Strain is a term originating from mechanics that is used to describe the deformation of materials, including myocardial tissue. Its measurement in echocardiography is based on myocardial tracking to evaluate the lengthening and shortening of its fibers during the cardiac cycle.^4^ In cardiology, myocardial strain assessment has been utilized to detect subclinical myocardial impairment, as its reduction frequently precedes a decline in ejection fraction and exhibits lower interobserver variability.^4^, ^5^ In this study, we analyzed myocardial deformation of cardiac chambers through transthoracic echocardiography with longitudinal and circumferential strain techniques in individuals with temporal lobe epilepsy (TLE) compared to a healthy control group.

## 2 MATERIALS AND METHODS

Forty‐three individuals >18 years old with TLE from the reference center for the comprehensive care of epilepsy outpatient clinic of the Federal University of Santa Catarina were consecutively included from July 2022 to November 2023. The diagnostic criteria and classification of epilepsy were based on the International League Against Epilepsy (ILAE).^6^ The control group consisted of volunteers without a diagnosis of epilepsy, matched by sex, age, and body mass index (BMI).

All individuals were interviewed based on a standardized protocol that included screening of cardiac risk factors. Additionally, they were submitted to an extensive clinical examination, comprising cardiac and pulmonary auscultation, and peripheral pulse palpation.

Echocardiography was performed in a standardized manner using Philips EPIQ CVx 3D echocardiography equipment with a S5‐1 multifrequency sector transducer (Philips Ultrasound). Cardiac chamber dimensions, left ventricular (LV) mass and ejection fraction (LVEF) measured by Simpson's method, and right ventricular (RV) function parameters comprising the tricuspid annular plane systolic excursion and tricuspid annulus systolic excursion velocity using tissue Doppler were obtained as described earlier.^7^ Automated cardiac motion quantification (aCMQ) software was used to analyze longitudinal and circumferential strain.

LV longitudinal strain (LVLS) analysis was performed using the apical four‐, three‐, and two‐chamber views focused on the left ventricle. LV global longitudinal strain (LVGLS) represents the mean value of all views. RV free wall longitudinal strain (RVFWLS) analysis was performed in the apical four‐chamber view focused on the right ventricle. Short‐axis cross‐sectional views (basal, mid, and apical) were used for circumferential strain. Right atrial (RA) strain was analyzed in the apical four‐chamber view, and left atrial (LA) strain was analyzed in the apical four and two‐chamber views (Supplementary Files 1 and 2).

Continuous variables were presented as mean ± SD and categorical variables as percentage of the total. The normal distribution of data was tested by the Kolmogorov–Smirnov test. The two‐tailed Student *t*‐test was used to compare quantitative variables, and Fisher exact test or Pearson chi‐squared test was used to compare categorical variables and frequency of occurrence. Hierarchical multiple linear regression was performed to explore independent predictor variables of global longitudinal and circumferential strain values of the LV, atrial, and RV strain. Independent variables related to clinical, demographic, and echocardiographic data identified by univariate analysis with a *p*‐value < .1 were selected for the final multiple linear regression model. Statistical analysis was performed using IBM SPSS for Windows, version 22, and Microsoft Excel for Microsoft 365 MSO (version 2211) software. Significance level was defined as ≤5% (*p* ≤ .05).

This study was approved by the research ethics committee of the Federal University of Santa Catarina (No. 5.425.300). Informed consent form was obtained from all participants.

## 3 RESULTS

### 3.1 Demographics

Eighty‐six individuals were consecutively included: 43 patients with TLE matched for age, sex, and BMI with 43 controls without epilepsy. Clinical characteristics and cardiovascular risk factors were similar in both groups, except for physical activity practice, which was lower among PWE (Table 1). Treatment with more than one antiseizure medication occurred in 74% of PWE, with carbamazepine being the most prescribed medication (Supplementary File 3).

**TABLE 1:** Clinical, demographic, and echocardiographic parameters of the two groups.

Parameter	Epilepsy, *n* = 43	Controls, *n* = 43	*p*
Clinical characteristics			
Age, years	46.05 ± 12.63	46.30 ± 12.47	.925
Sex, male	23 (53.5%)	23 (53.5%)	1.00
Hypertension	10 (23.25%)	10 (23.25%)	1.00
Dyslipidemia	14 (32.55%)	10 (23.25%)	.336
Diabetes	2 (4.65%)	0	.152
Smoking	4 (9.30%)	6 (13.95%)	.501
Physical activity practice^a^	10 (23.25%)	23 (53.48%)	.**003**
Epilepsy data			
Etiology	Mesial temporal sclerosis (91%) Cryptogenic (7%) Neoplasia (2%)	N/A	
Age at onset of epilepsy, years	14.4 ± 11.85		
Epilepsy duration, years	31.6 ± 13.83		
Structural lesion lateralization per MRI	Right‐sided, 21 (48.8%)		
Number of ASMs per individual	2.2 ± .98		
Individuals in polytherapy	32 (74.4%)		
Epilepsy surgery	7 (16.3%)		
Echocardiogram			
LVEF with Simpson's method	63.50 ± 5.30	66.95 ± 3.45	.**001**
TAPSE	22.59 ± 3.51	25.01 ± 3.32	.**001**
RV S'	11.90 ± 1.88	13.57 ± 1.75	**<.001**
LV AP2C strain	19.89 ± 2.94	20.60 ± 2.35	.217
LV AP3C strain	19.30 ± 2.70	20.94 ± 2.19	.**002**
LV AP4C strain	19.42 ± 2.94	20.77 ± 2.55	.**025**
LV global strain	19.54 ± 2.39	20.76 ± 1.95	.**011**
LV base circumferential strain	27.07 ± 5.72	25.66 ± 4.12	.200
LV mid circumferential strain	27.27 ± 5.05	29.21 ± 4.07	.056
LV apical circumferential strain	30.81 ± 9.89	33.01 ± 6.98	.242
LV global circumferential strain	28.09 ± 5.58	29.29 ± 3.23	.230
RV free wall strain	20.39 ± 4.17	22.36 ± 3.62	.**024**
Biplane LA reservoir strain	34.68 ± 7.18	38.20 ± 9.25	.052
Biplane LA conduit strain	20.64 ± 5.87	21.84 ± 7.02	.389
Biplane LA contractile strain	13.92 ± 4.43	16.31 ± 3.86	.**009**
RA reservoir strain	33.39 ± 9.03	41.01 ± 8.43	.**0001**
RA conduit strain	21.45 ± 8.46	25.16 ± 6.40	.**025**
RA contractile strain	12.15 ± 4.21	15.85 ± 4.88	.**0003**

Abbreviations: AP2C, apical two‐chamber view; AP3C, apical three‐chamber view; AP4C, apical four‐chamber view; ASM, antiseizure medication; LA, left atrial; LV, left ventricle; LVEF, LV ejection fraction; MRI, magnetic resonance imaging; N/A, not applicable; RA, right atrial; RV, right ventricle; RV S', tricuspid annulus systolic excursion velocity using pulsed tissue Doppler; TAPSE, tricuspid annular plane systolic excursion.

^a^ At least two times per week.

### 3.2 Echocardiogram

Cardiac chamber dimensions and LV diastolic parameters were similar in both groups. No individual in the control group had altered LV geometry, whereas five PWE exhibited altered geometry (*p* = .021). LVEF and RV function variables were lower among PWE (Table 1).

### 3.3 Myocardial strain

PWE had lower LV longitudinal strain values in the apical four‐ and three‐chamber view and in the global average value. RVFWLS was lower among PWE compared to controls. For the LA, biplanar contractile strain was significantly lower in PWE, and biplanar reservoir strain, although lower in PWE, reached borderline significance (*p* = .052). For the right atrium, reservoir, conduit, and contractile strain values were significantly lower in PWE. There was no significant difference between groups when comparing LV circumferential strain values (Table 1).

### 3.4 Multivariate analysis

Epilepsy was an independent predictor of reduced LA biplanar reservoir longitudinal strain (*p* = .009), LA biplanar contractile longitudinal strain (*p* = .003), RA reservoir strain (*p* = .021), and RA contractile strain (p = .003; Supplementary File 4).

### 3.5 Multivariate analysis in the epilepsy group

Polytherapy with antiseizure medications was an independent predictor of reduced LVGLS (*p* = .009; Supplementary File 5).

Epilepsy duration was an independent predictor of reduced RA reservoir (*p* <.001) and contractile strain (*p* = .008) and of reduced LA biplanar reservoir strain (*p* = .004; Supplementary File 5).

## 4 DISCUSSION

**FIGURE 1:**
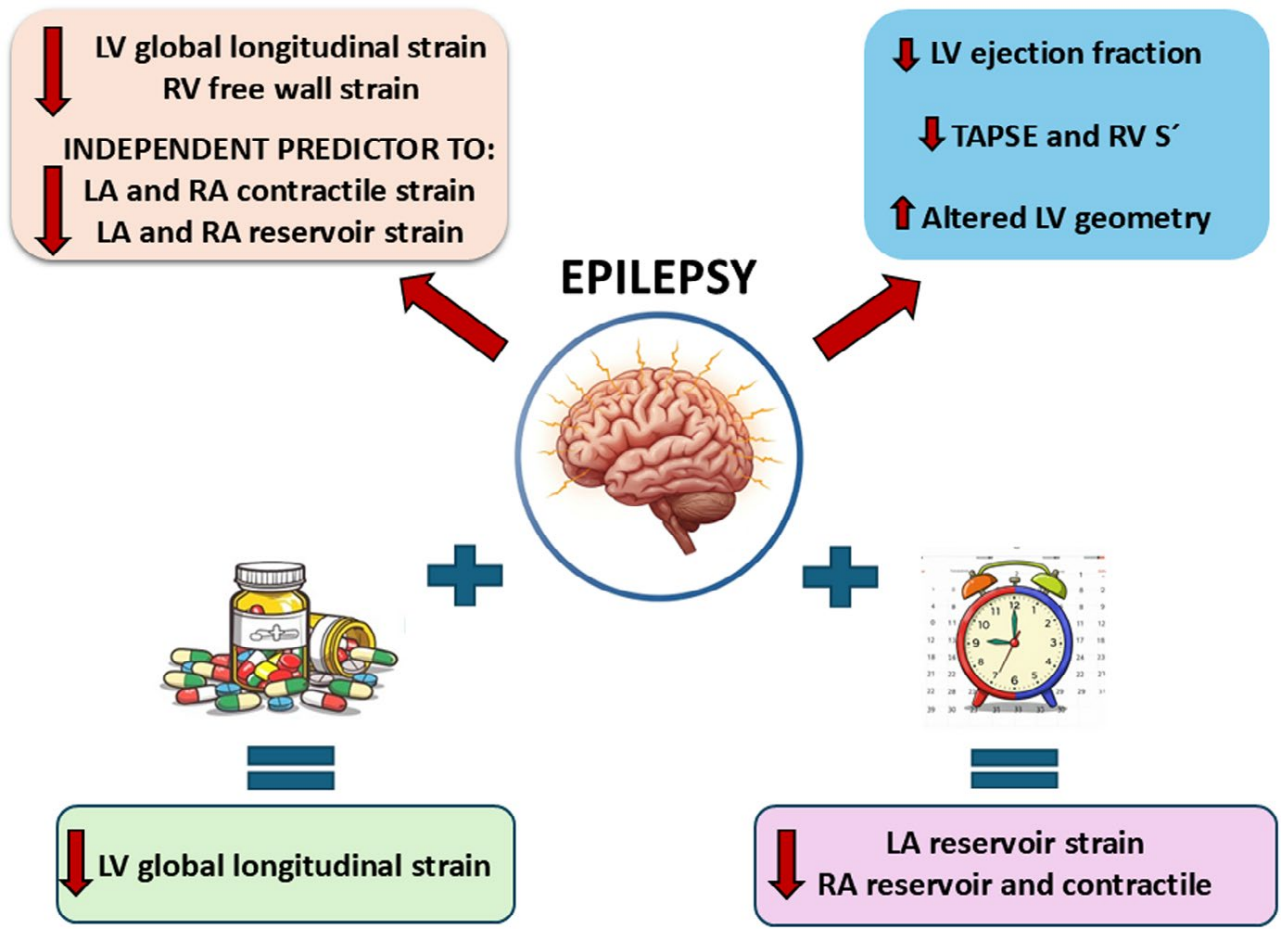
People with epilepsy had reduced left ventricular (LV) global longitudinal strain (LVGLS) (*p* = .011), right ventricular (RV) free wall longitudinal strain (*p* = .024), LV ejection fraction (*p* = .001), and RV function parameters (tricuspid annular plane systolic excursion [TAPSE] (*p* = .001) and tricuspid annulus systolic excursion velocity using pulsed tissue Doppler [RV S’] (*p* < .001)) and increased number of participants with altered LV geometry (*p* = .021), compared to an age‐, sex‐, and body mass index‐matched control group. Epilepsy was an independent predictor of reduced reservoir (*p* = .009 for LA and *p* = .021 for RA) and contractile strain of both atria (*p* = .003 for both LA and RA). Polytherapy with antiseizure medication was an independent predictor of reduced LVGLS (*p* = .009). Epilepsy duration was an independent predictor of reduced left atrial (LA) biplanar reservoir strain (*p* = .004) and reduced right atrial (RA) reservoir (*p* < .001) and contractile strain (*p* = .008).

We investigated subtle and early changes in cardiac function in PWE with a diagnosis of TLE according to the ILAE, compared to a control group without epilepsy. Although the study population consisted of young individuals without a history of known cardiovascular disease, PWE had lower strain values in all four cardiac chambers (Figure 1 and Supplementary File 6).

Poorly controlled and recurrent epileptic seizures over time expose the cardiovascular system to hypoxemia and catecholaminergic toxicity, leading to subtle and progressive damage.^3^ In initial phases, traditional measures of cardiac function, such as LVEF, may still be normal.^5^ In the present study, ventricular contractile function, although lower in PWE, was still within the normal range.

Longitudinal strain by speckle tracking has been shown to be more sensitive for detecting early and subclinical cardiac involvement, in addition to having lower inter‐ and intraobserver variability compared to LVEF.^5^ Lower LVGLS value has been associated with ventricular arrhythmia in individuals with preserved ejection fraction,^8^ and it is an independent predictor of ejection fraction decline and future heart failure development.^9^ In the Copenhagen City Heart Study, 1296 healthy individuals were followed for an average of 11 years. In this population, a 1% reduction in LVGLS was related to a 12% increase in the risk of heart failure, myocardial infarction, and cardiac death.^10^ In the Cardiovascular Health Study, among 3313 individuals followed for an average of 10 years, for each 1‐SD decrease in LVGLS there was a 25% increase in the risk of coronary artery disease.^11^

Cardiac strain assessment is not entirely new in epilepsy. Çelik et al., found lower LVGLS values among children with well‐controlled epilepsy on monotherapy.^12^ Schreiber et al., evaluating children with refractory epilepsy, found a reduction in LV longitudinal strain compared to a control group. In that study, however, only the apical four‐chamber view, and not global longitudinal strain analysis, was assessed. Regarding circumferential strain analysis, these authors, like Çelik et al., used only the short‐axis view at the papillary level.^13^ Zaki et al., also studying children, found lower myocardial deformation values of RV and LV among patients with refractory and well‐controlled epilepsy compared to a control group.^14^ Only one small study evaluated LV strain in adults. The authors could not find a significant difference in myocardial deformation between cases and controls due to the small sample size (they analyzed LVGLS of only five patients with epilepsy).^15^

Although PWE had lower LVGLS and RVFWLS values, LV circumferential strain was similar between the two groups. Longitudinal strain corresponds to myocardial fibers located just below the endocardium. This region is subject to greater hemodynamic stress and ischemia and is more sensitive to ventricular geometry changes and thus affected earlier than circumferential strain. Adaptively, circumferential strain tends to compensate for the loss of longitudinal strain to maintain the LVEF.^4^

No previous study had investigated atrial strain in patients with epilepsy. Atrial strain assessment has gained importance for its contribution to diagnosing heart failure with preserved ejection fraction (HFpEF) and cardiac arrhythmias.^4^, ^16^

The present study found a lower contractile strain value in the four‐chamber view, lower reservoir strain and contractile strain value in the two‐chamber view, and lower biplanar contractile strain value of the left atrium in PWE. Furthermore, lower RA strain values in the reservoir, conduit, and contractile phases were seen among PWE.

Park et al., analyzing a cohort of 4312 individuals for 5 years, after hospitalization for heart failure, showed that a lower LA reservoir strain value increased the risk of developing future atrial fibrillation (AF) by 72%. Furthermore, each 1% increase in LA reservoir strain was associated with a 3% decreased risk of AF.^16^

The risk of cardiac arrhythmias in patients with epilepsy, including AF, has been the subject of epidemiological and electrophysiological studies. Recently, Fialho et al. demonstrated that PWE had P‐wave heterogeneity equal to a nearly 20 years older paroxysmal AF population without epilepsy.^17^

Furthermore, searching for markers related to the risk of heart failure, such as ventricular strain and atrial strain (the latter associated with HFpEF), may be necessary in epilepsy. Doege et al., evaluating a cohort involving 9165 individuals with epilepsy compared with controls, found a 56% higher incidence of heart failure in PWE in 10 years of follow‐up.^18^

The influence of polytherapy and disease duration on strain values may indicate that more severe forms of the disease and longer exposure to epilepsy itself and/or antiseizure medications are related to more significant myocardial damage.^3^

Self‐reported physical activity (measured as a categorical variable: active or inactive) was less than half in the epilepsy group compared to controls (Table 1). Despite that, as shown in the supplementary material, our statistical analysis did not demonstrate a significant correlation between self‐reported physical activity and the difference in strain values between the epilepsy and control groups (Supplementary File 4). Among individuals with epilepsy, physical activity was significantly correlated with an increased RA contractile strain value (Supplementary File 5).

A clinical syndromic approach for the diagnosis of the “epileptic heart” was proposed by Verrier et al.^19^ In their article, the authors pointed out that increased LV stiffness, elevated LV filling pressure, and increased LA volume could be used as echocardiographic markers for the “epileptic heart.”^19^ Our findings regarding lower strain in all cardiac chambers may add to those criteria.

This study has some limitations. Philips aCMQ software dedicated to the LV was used for all strain analysis. Although we did not use specific strain software for the other cardiac chambers, this adaptation is commonly used. This was a single‐center study, so these data should ideally be reproduced at other centers. Due to trial design, hard outcomes and their relation to strain were not evaluated.

## 5 CONCLUSIONS

In conclusion, PWE, compared to a control group had altered LV geometry, LVEF, RV contractility and lower atrial and ventricular strain values. Our findings suggest that PWE, even without apparent heart disease, may have subclinical myocardial dysfunction. These changes may help explain this population's increased risk of cardiac events. Additional studies are needed to determine the clinical implications of these findings.

## AUTHOR CONTRIBUTIONS

Ramsés Miotto conceptualized and designed the study, assisted with data collection, and wrote the manuscript. Márcia Tatsch Cavagnollo assisted with data collection and revision of the manuscript. Katia Lin assisted with conceptualizing, data collection, and revising the manuscript. Guilherme L. Fialho conceptualized and designed the study and revised the manuscript.

## CONFLICT OF INTEREST STATEMENT

K.L. holds a National Council for Scientific and Technological Development–CNPq PQ1C Research Fellowship (Process Number 306916/2023‐1). The remaining authors have no conflicts of interest.

## ETHICS STATEMENT

This study was approved by the research ethics committee of the Federal University of Santa Catarina through the Plataforma Brasil (No. 5.425.300) on May 23, 2022. We confirm that we have read the Journal's position on issues involved in ethical publication and affirm that this report is consistent with those guidelines.

## Supporting information


**Data S1.** Supporting information.

## Data Availability

Additional data for this study will be available from the corresponding author upon reasonable request.

## References

[1] Beghi E . The epidemiology of epilepsy. Neuroepidemiology. 2020;54(2):185–191. 10.1159/000503831 31852003

[2] Bardai A , Lamberts RJ , Blom MT , Spanjaart AM , Berdowski J , van der Staal SR , et al. Epilepsy is a risk factor for sudden cardiac arrest in the general population. PLoS One. 2012;7(8):e42749. 10.1371/journal.pone.0042749 22916156 PMC3419243

[3] Verrier RL , Pang TD , Nearing BD , Schachter SC . The epileptic heart: concept and clinical evidence. Epilepsy Behav. 2020;105:106946. 10.1016/j.yebeh.2020.106946 32109857

[4] Smiseth OA , Rider O , Cvijic M , Valkovič L , Remme EW , Voigt JU . Myocardial strain imaging: theory, current practice, and the future. JACC Cardiovasc Imaging. 2025;18(3):340–381. 10.1016/j.jcmg.2024.07.011 39269417

[5] Potter E , Marwick TH . Assessment of left ventricular function by echocardiography: the case for routinely adding global longitudinal strain to ejection fraction. JACC Cardiovasc Imaging. 2018;11(2 Pt 1):260–274. 10.1016/j.jcmg.2017.11.017 29413646

[6] Fisher RS , Cross JH , French JA , Higurashi N , Hirsch E , Jansen FE , et al. Operational classification of seizure types by the international league against epilepsy: position paper of the ILAE Commission for Classification and Terminology. Epilepsia. 2017;58(4):522–530. 10.1111/epi.13670 28276060

[7] Fialho GL , Pagani AG , Wolf P , Walz R , Lin K . Echocardiographic risk markers of sudden death in patients with temporal lobe epilepsy. Epilepsy Res. 2018;140:192–197. 10.1016/j.eplepsyres.2018.01.016 29414527

[8] Yoshida Y , Jin Z , Nakanishi K , Matsumoto K , Homma S , Mannina C , et al. Subclinical left ventricular dysfunction and ventricular arrhythmias in older adults with Normal ejection fraction. J Am Heart Assoc. 2023;12(16):e030274. 10.1161/JAHA.123.030274 37577940 PMC10492955

[9] Haji K , Huynh Q , Wong C , Stewart S , Carrington M , Marwick TH . Improving the characterization of stage a and B heart failure by adding global longitudinal strain. JACC Cardiovasc Imaging. 2022;15(8):1380–1387. 10.1016/j.jcmg.2022.03.007 35926896

[10] Biering‐Sørensen T , Biering‐Sørensen SR , Olsen FJ , Sengeløv M , Jørgensen PG , Mogelvang R , et al. Global longitudinal strain by echocardiography predicts long‐term risk of cardiovascular morbidity and mortality in a low‐risk general population: the Copenhagen City Heart Study. Circ Cardiovasc Imaging. 2017;10(3):e005521. 10.1161/CIRCIMAGING.116.005521 28264868 PMC5363277

[11] Massera D , Hu M , Delaney JA , Bartz TM , Bach ME , Dvorak SJ , et al. Adverse cardiac mechanics and incident coronary heart disease in the cardiovascular health study. Heart. 2022;108(7):529–535. 10.1136/heartjnl-2021-319296 34257074 PMC8755845

[12] Çelik SF , Baratalı E , Güven AS , Torun YA . Left ventricular myocardial deformation abnormalities in seizure‐free children with epilepsy. Seizure. 2018;61:153–157. 10.1016/j.seizure.2018.08.017 30170299

[13] Schreiber JM , Frank LH , Kroner BL , Bumbut A , Ismail MO , Gaillard WD . Children with refractory epilepsy demonstrate alterations in myocardial strain. Epilepsia. 2020;61(10):2234–2243. 10.1111/epi.16652 33053223 PMC8191539

[14] Zaki ER , Mohamed LA , Agiba NA , Abo S , Mohammed A , Ismail M , et al. Relation between seizure control and ventricular functions in epileptic children. NeuroQuantology. 2022;20(4):1098–1101. 10.48047/NQ.2022.20.4.NQ22334

[15] González A , Haugaa KH , Brekke PH , Hopp E , Ørn S , Alvestad S , et al. Cardiac structure and function in epilepsy patients with drug‐resistant convulsive seizures. Case Rep Neurol. 2022;14(1):88–97. 10.1159/000522237 35431877 PMC8958580

[16] Park JJ , Park JH , Hwang IC , Park JB , Cho GY , Marwick TH . Left atrial strain as a predictor of new‐onset atrial fibrillation in patients with heart failure. JACC Cardiovasc Imaging. 2020;13(10):2071–2081. 10.1016/j.jcmg.2020.04.031 32682715

[17] Fialho GL , Pang TD , Kong WY , Tran AP , Yu CG , Rodriguez ID , et al. Individuals with chronic epilepsy have elevated P‐wave heterogeneity comparable to patients with atrial fibrillation. Epilepsia. 2023;64(9):2361–2372. 10.1111/epi.17686 37329175

[18] Doege C , Luedde M , Kostev K . Epilepsy is associated with an increased incidence of heart failure diagnoses. Epilepsy Behav. 2021;125:108393. 10.1016/j.yebeh.2021.108393 34731722

[19] Verrier RL , Pang TD , Nearing BD , Schachter SC . Epileptic heart: a clinical syndromic approach. Epilepsia. 2021;62(8):1780–1789. 10.1111/epi.16966 34236079

