# Supplementary material for: Strain analysis of cardiac chambers in individuals with temporal lobe epilepsy
Source: Epilepsia. 2025 Jun 13;66(8):e174–80. doi: 10.1111/epi.18472 (PMC12371644; doi:10.1111/epi.18472)
Supplement: Supplementary file 1 — Data S1. Supporting information. [file EPI-66-e174-s001.docx]

**SUPPLEMENTARY FILE 1** – Left ventricular longitudinal and circumferential strain analysis

| 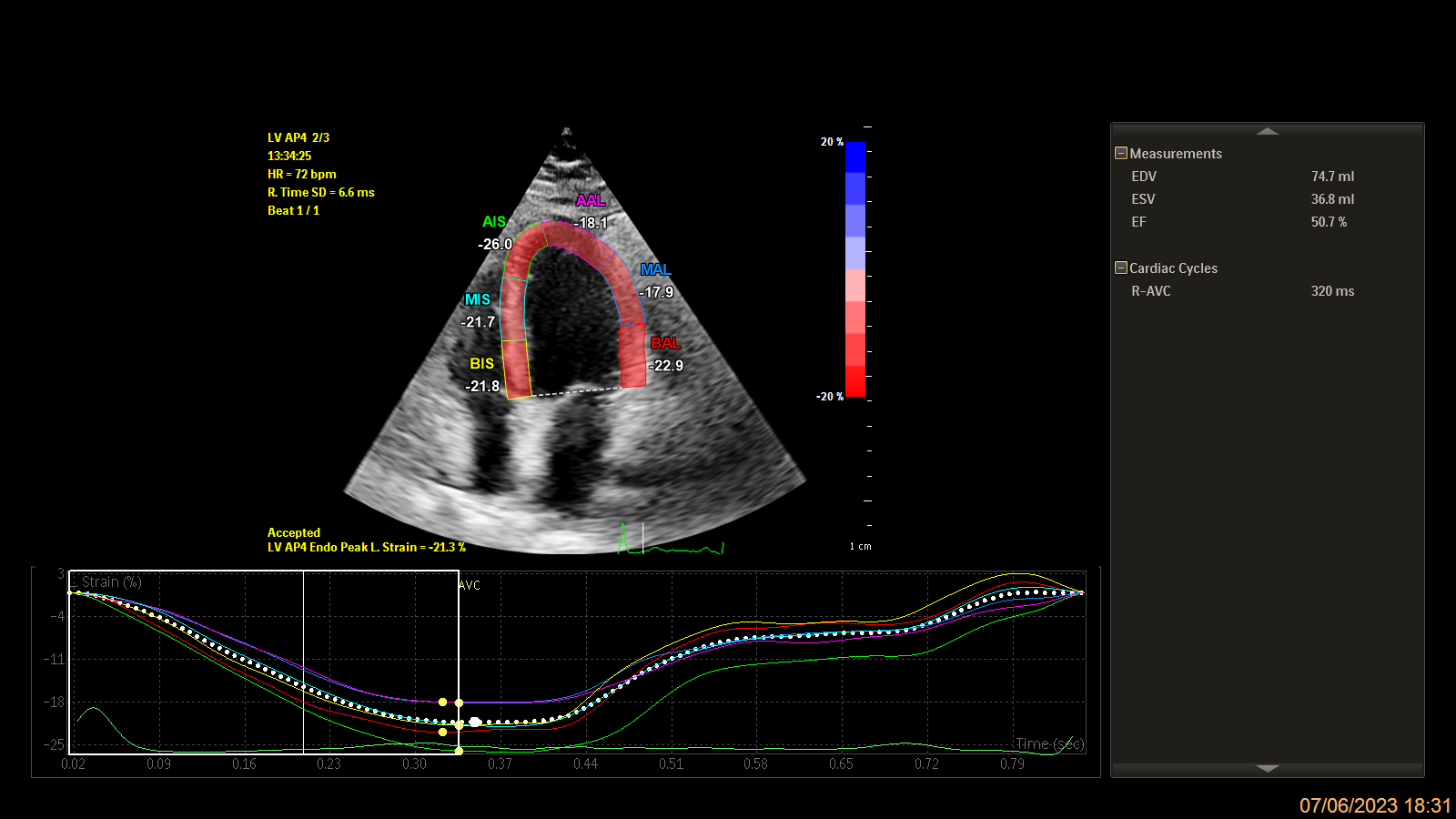 | 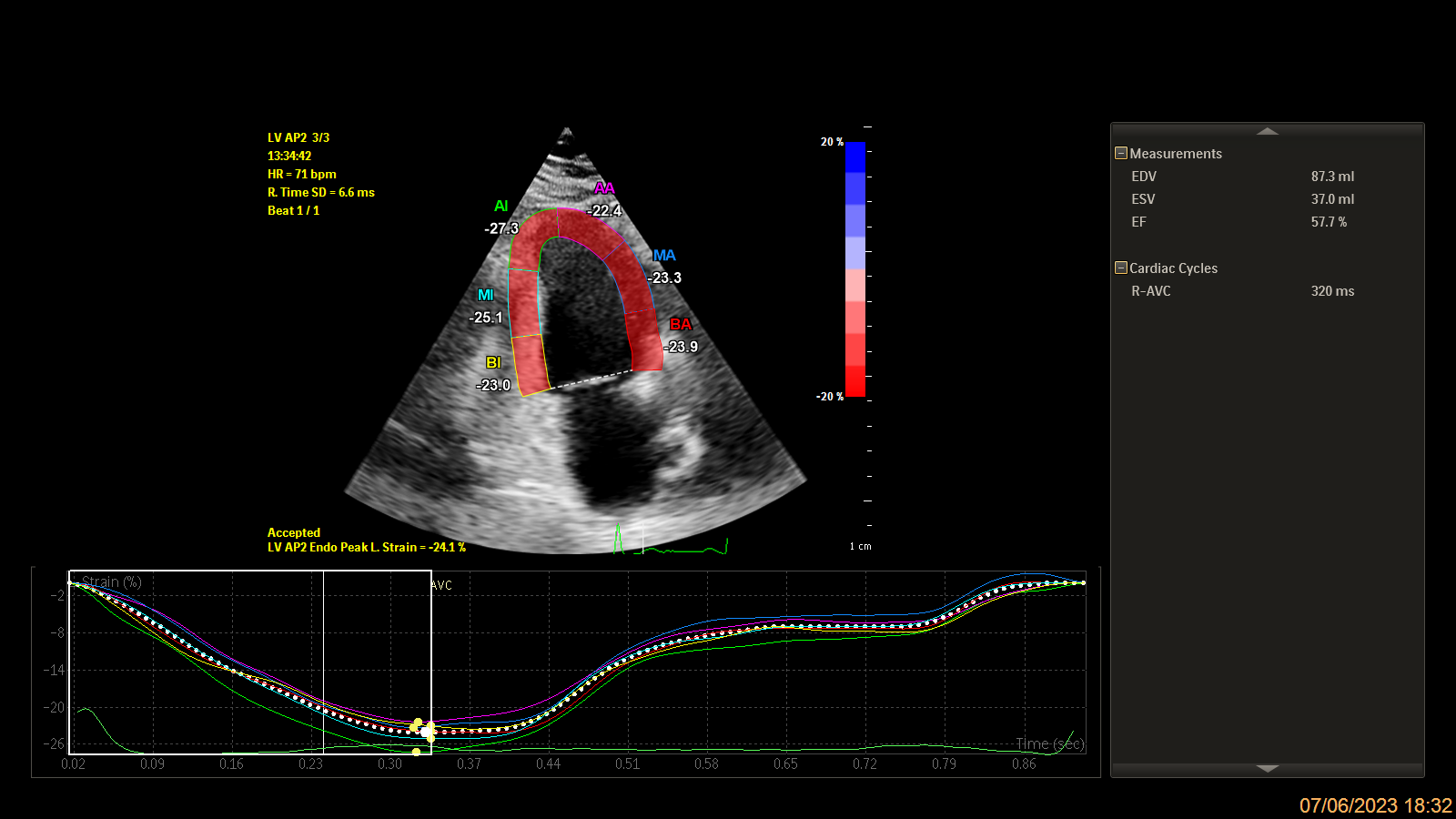 |
| --- | --- |
| LV four-chamber view longitudinal strain | LV two-chamber view longitudinal strain |
| 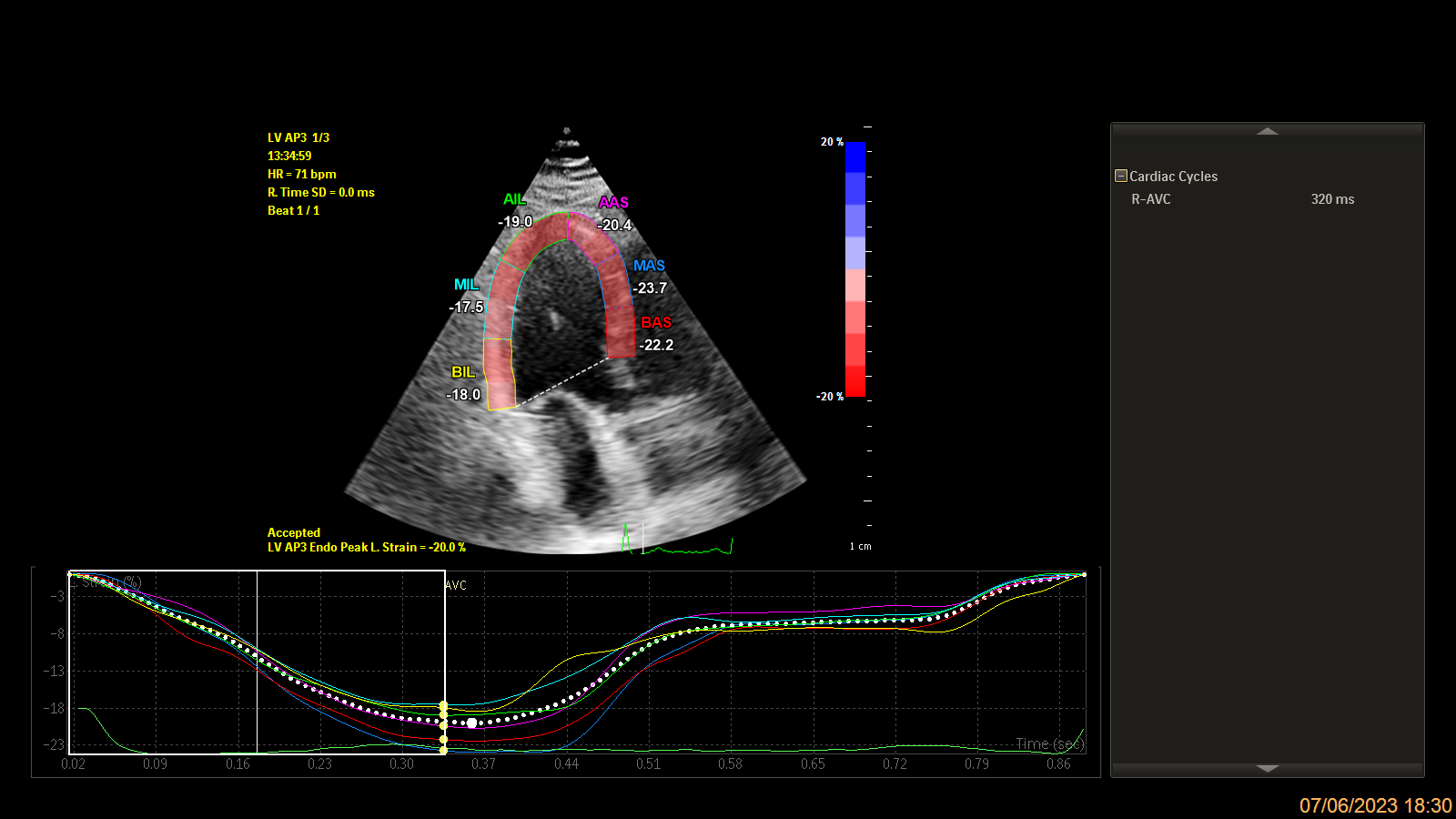 | 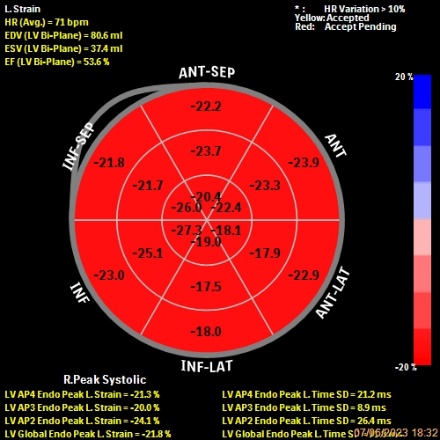 |
| LV three-chamber view longitudinal strain | LV global longitudinal strain (bull´s eye) |
| 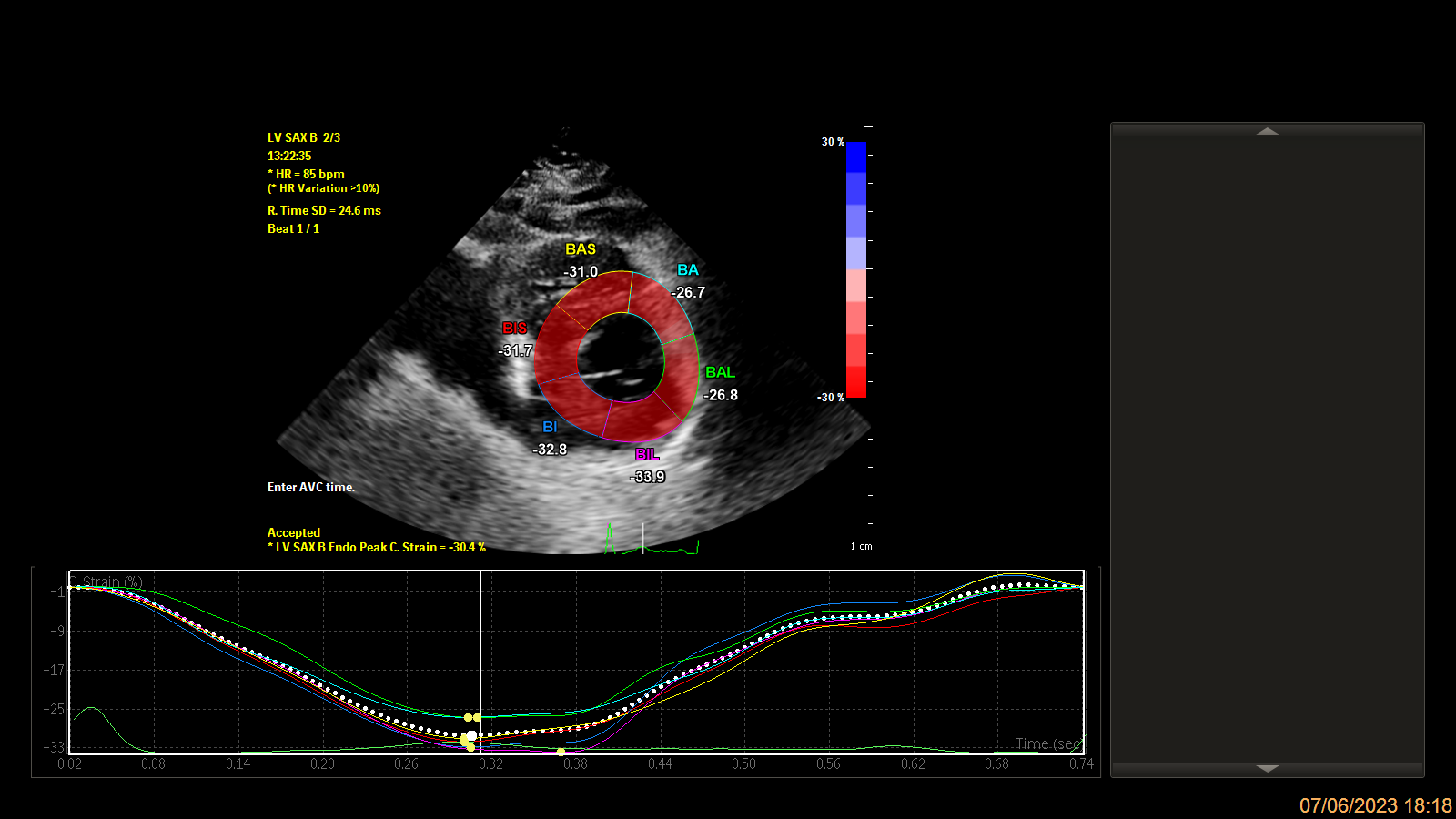 | 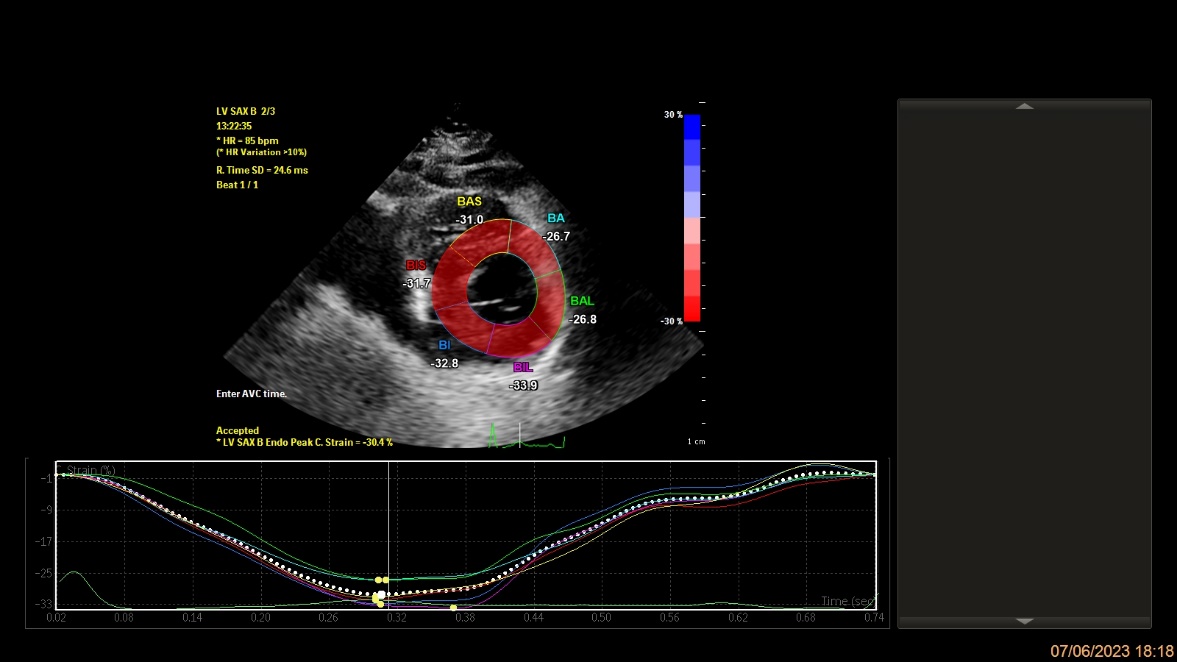 |
| LV basal circumferential strain | LV mid circumferential strain |
| 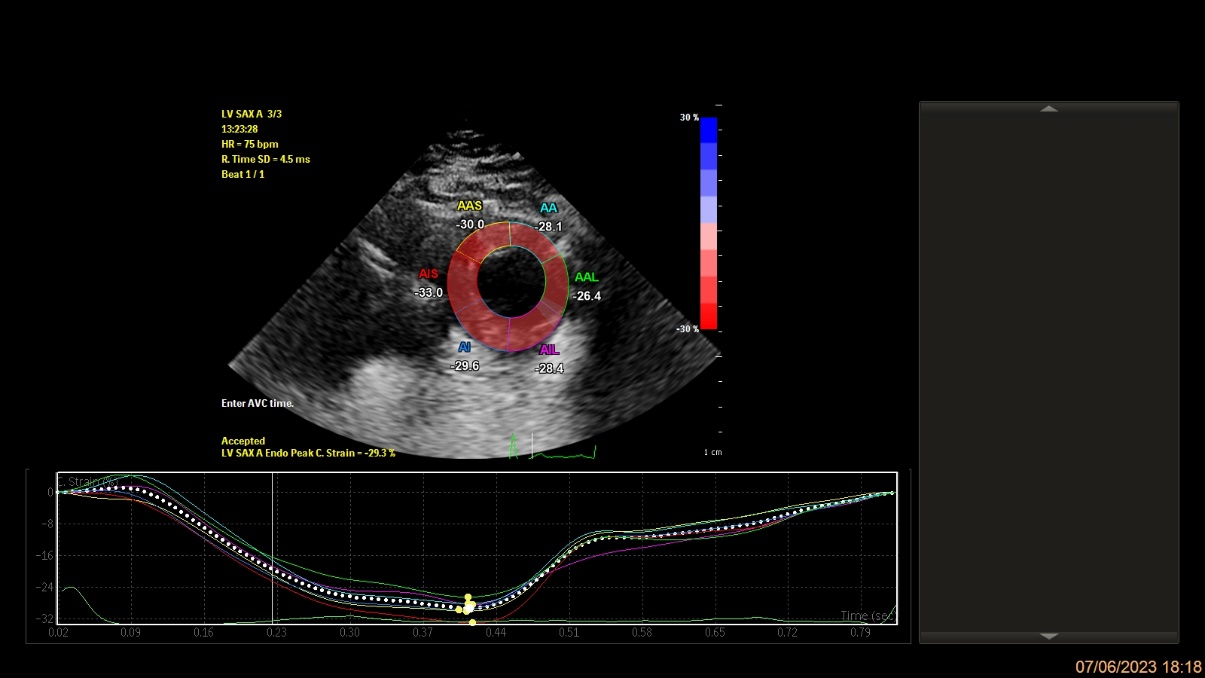 | 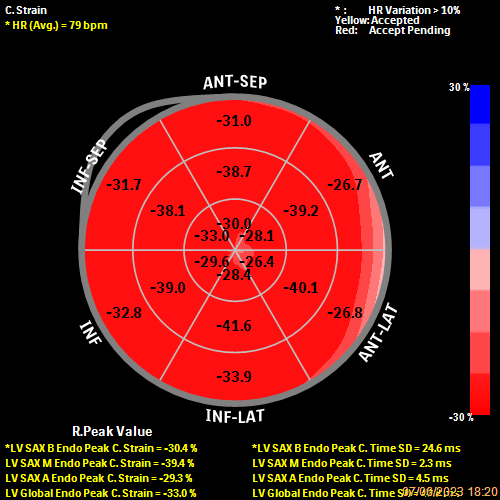 |
| LV apical circumferential strain | LV global circumferential strain (bull´s eye) |

**SUPPLEMENTARY FILE 1**: Strain corresponds to the myocardium shortening relative to its initial dimension. In echocardiography, shortening in the longitudinal and circumferential directions are expressed as a negative percentage. In the apical four-chamber view, the inferior septal and anterolateral walls are assessed; in the apical two-chamber view, the anterior and inferior walls are assessed; and in the apical three-chamber view, the inferolateral and anteroseptal walls are assessed. The sum of the longitudinal strain of all LV segments results in a graphical representation called “bull's eye” with a corresponding mean global value. Circumferential strain is performed in basal, mid, and apical short-axis views of the left ventricle, and the sum of the strain of its segments results in a bull's eye graph with a mean global value. LV – Left ventricle

**SUPPLEMENTARY FILE 2** – Left atrial, right atrial and right ventricular strain

| 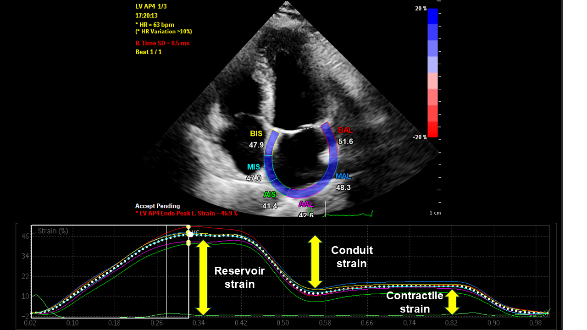 | |
| --- | --- |
| LA four-chamber view strain | |
| 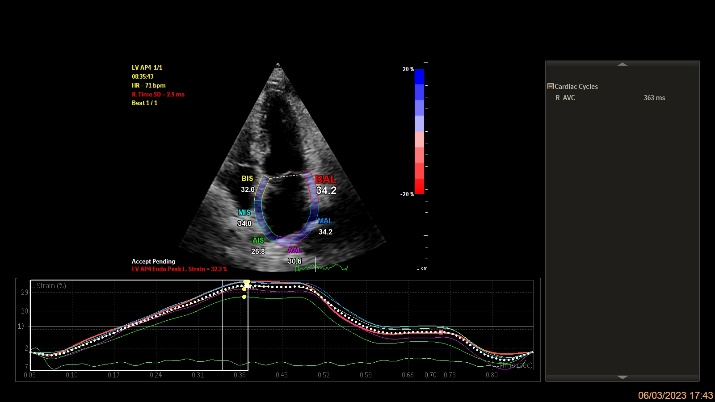 | 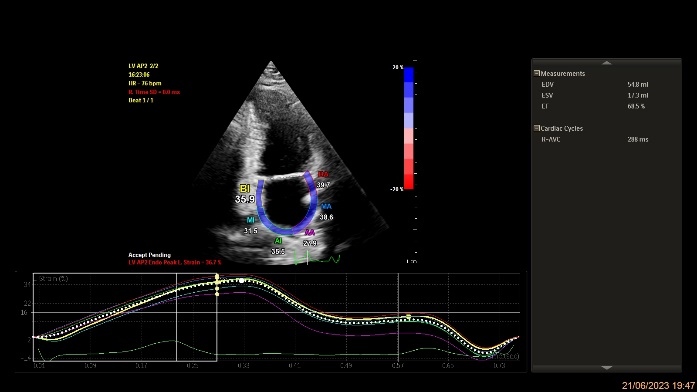 |
| LA four-chamber view strain | LA two-chamber view strain |
| 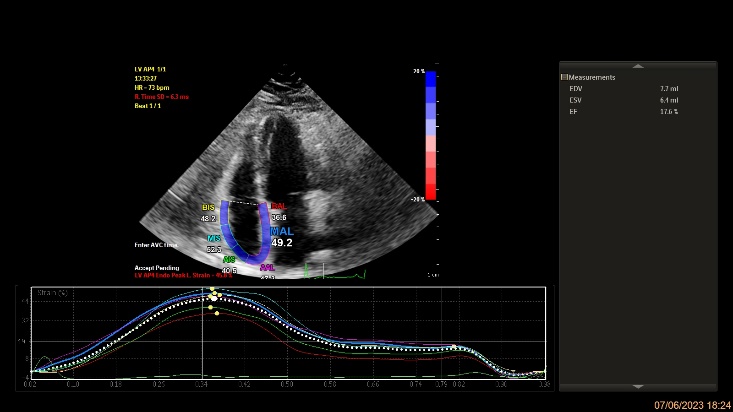 | 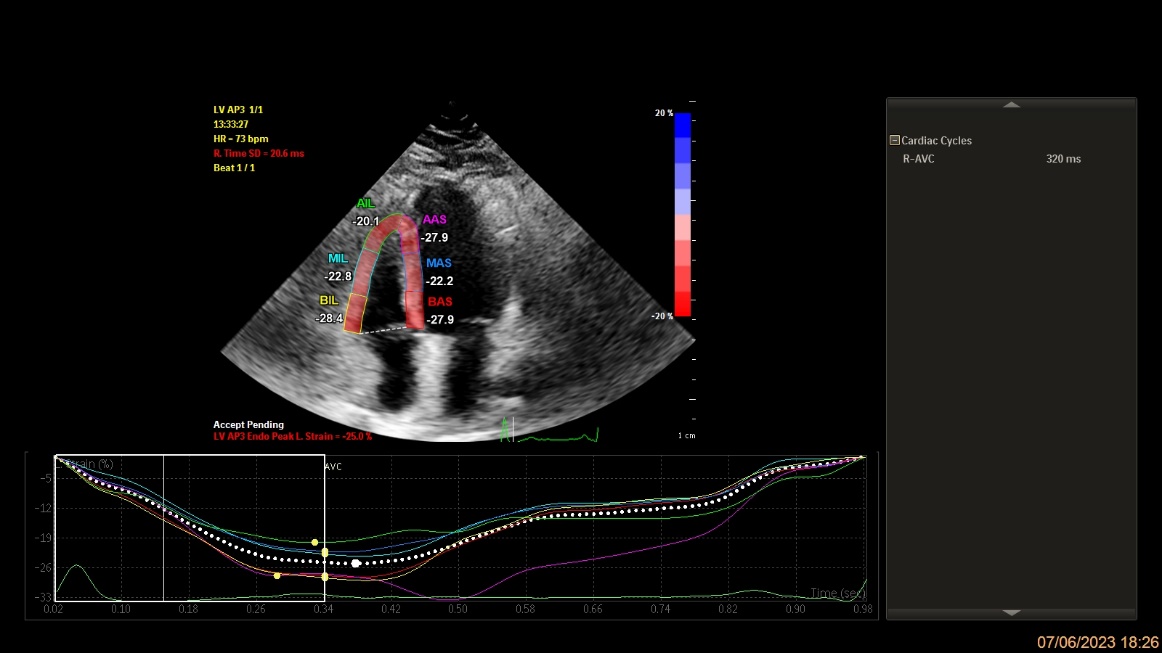 |
| RA four-chamber view strain | RV free wall longitudinal strain |

**SUPPLEMENTARY FILE 2**: Left atrial strain was analyzed in the apical four and two-chamber views, and right atrial strain was analyzed in the apical four-chamber view. The QRS complex (synchronized to the echocardiogram) was used to identify the onset of the atrial cycle. Atrial strain is analyzed in each phase of the cardiac cycle. Reservoir strain corresponds to myocardial deformation during left atrial expansion (ventricular systole), conduit strain corresponds to the value during early diastole (passive ventricular filling), and contractile strain refers to the value during late ventricle diastole where atrial contraction occurs (active ventricular filling). Right ventricular (RV) free wall longitudinal strain (opposite wall from the interventricular septum) was obtained from the apical four-chamber view (values related to the interventricular septum are ignored for the RV strain and considered for the LV strain). LA – Left atrium; RA – Right atrium; RV – Right ventricle.

| Antiseizure medication: | Percentage of individuals treated with |
| --- | --- |
| Carbamazepine | 26 (60.46%) |
| Phenobarbital | 19 (44.18%) |
| Levetiracetam | 13 (30.23%) |
| Clobazam | 11 (25.58%) |
| Lamotrigine | 7 (16.27%) |
| Oxcarbazepine | 4 (9.30%) |
| Valproic acid | 4 (9.30%) |
| Topiramate | 4 (9.30%) |
| Lacosamide | 3 (6.97%) |
| Phenytoin | 3 (6.97%) |
| Clonazepam | 3 (6.97%) |
| Lorazepam | 1 (2.32%) |

**Supplementary FILE 3 – Prescribed antiseizure medications**

**SUPPLEMENTARY FILE 4 – MULTIVARIATE ANALYSIS IN BOTH GROUPS (n = 86)**

| Model | AdjustedR^2^ | p* | Variables | B coefficient (CI 95%) | Standardized B^2^ | p |
| --- | --- | --- | --- | --- | --- | --- |
| LVGLS^1^ | 0.297 | **<0.001** | Constant | 12.26 (5.34 a 19.17) | n/a** | **<0.001** |
|  | | | LVEF (Simpson) | 0.19 (0.11 a 0.28) | 0.410 | **<0.001** |
|  |  |  | Diastolic BP | -0.06 (-0.09 a -0.02) | -0.302 | **0.002** |
| LARS^2^ | 0.258 | **<0.001** | Constant | 52.08 (45.85 a 58.31) | n/a | **<0.001** |
|  | | | Age | -0.30 (-0.43 a -0.17) | -0.444 | **<0.001** |
|  |  |  | **Epilepsy** | -4.25 (-7.40 a -1.10) | -0.254 | **0.009** |
|  |  |  | DM | 14.26 (3.76 a 24.75) | 0.257 | **0.008** |
| LACS^3^ | 0.200 | **<0.001** | Constant | 3.84 (-1.34 a 9.02) | n/a | 0.144 |
|  | | | **Epilepsy** | -2.48 (-0.89 a -4.07) | -0.312 | **0.003** |
|  |  |  | Heart rate | 0.11 (0.03 a 0.19) | 0.285 | **0.006** |
| RARS^4^ | 0.442 | **<0.001** | Constant | 27.66 (-4.35 a 59.67) | n/a | 0.089 |
|  | | | LVEF (Simpson) | 0.47 (0.11 a 0.83) | 0.238 | **0.012** |
|  |  |  | TAPSE | 0.93 (0.45 a 1.41) | 0.356 | **0.001** |
|  |  |  | Systolic BP | -0.11 (-0.20 a -0.03) | -0.232 | **0.010** |
|  |  |  | RA volume | -0.26 (-0.43 a -0.09) | -0.284 | **0.003** |
|  |  |  | Heart rate | -0.25 (-0.41 a -0.09) | -0.275 | **0.003** |
|  |  |  | **Epilepsy** | -4.19 (-7.72 a -0.66) | -0.222 | **0.021** |
| RACS^5^ | 0.239 | **<0.001** | Constant | 7.31 (0.34 a 14.27) | n/a | **0.040** |
|  | | | **Epilepsy** | -3.01 (-4.98 a -1.05) | -0.310 | **0.003** |
|  |  |  | Female sex | 2.71 (0.86 a 4.56) | 0.278 | **0.005** |
|  |  |  | TAPSE | 0.29 (0.020 a 0.56) | 0.217 | **0.036** |
| RVFWLS^6^ | 0.150 | **<0.001** | Constant | -9.54 (-25.12 a 6.04) | n/a | 0.227 |
|  | | | LVEF (Simpson) | 0.33 (0.09 a 0.56) | 0.281 | **0.008** |
|  |  |  | TAPSE | 0.38 (0.06 a 0.69) | 0.243 | **0.020** |
|  | ^1^ left ventricle global longitudinal strain; ^2^ left atrial reservoir strain; ^3^ left atrial contractile strain; ^4^ right atrial reservoir strain; ^5^ right atrial contractile strain; ^6^ right ventricle free wall longitudinal strain; LVEF: left ventricle ejection fraction; DM: diabetes mellitus; TAPSE: tricuspid annular plane systolic excursion; BP: blood pressure; RA: right atrium  * model´s p  ** not applicable | | | | | |

**SUPPLEMENTARY FILE 5 – MULTIVARIATE ANALYSIS IN EPILEPSY GROUP (n = 43)**

| Model | Adjusted R^2^ | | p* | Variables | B coefficient (IC 95%) | Standardized B^2^ | p |
| --- | --- | --- | --- | --- | --- | --- | --- |
| LVGLS^1^ | 0.325 | | **<0.001** | Constant | 17.91 (7.08 a 28.74) | n/a** | **0.002** |
|  | | | | Diastolic BP | -0.07 (-0.13 a -0.01) | -0.335 | **0.021** |
|  |  |  |  | **Polytherapy** | -1.91 (-3.32 a -0.50) | -0.352 | **0.009** |
|  |  |  |  | LVEF (Simpson) | 0.14 (0.01 a 0.27) | 0.309 | **0.034** |
| LARS^2^ | 0.298 | | **<0.001** | Constant | 50.90 (42.81 a 59.00) | n/a | **<0.001** |
|  | | | | **Epilepsy duration** | -0.21 (-0.35 a -0.07) | -0.397 | **0.004** |
|  |  |  |  | LA volume | -0.36 (-0.64 a -0.08) | -0.347 | **0.012** |
| LACS^3^ | 0.200 | | **0.003** | Constant | 3.99 (-2.12 a 10.11) | n/a | 0.194 |
|  | | | | Heart rate | 0.15 (0.05 a 0.24) | 0.448 | **0.003** |
| RARS^4^ | 0.459 | | **<0.001** | Constant | 63.98 (53.09 a 74.88) | n/a | **<0.001** |
|  | | | | LV mass | -0.27 (-0.39 a -0.15) | -0.521 | **<0.001** |
|  |  |  |  | **Epilepsy duration** | -0.31 (-0.47 a -0.15) | -0.455 | **<0.001** |
| RACS^5^ | 0.197 | | **0.006** | Constant | 15.08 (12.07 a 18.08) | n/a | **<0.001** |
|  | | | | **Epilepsy duration** | -0.12 (-0.21 a -0.03) | -0.400 | **0.008** |
|  |  |  |  | Physical activity | 3.35 (0.53 a 6.16) | 0.345 | **0.021** |
| RVFWLS^6^ | 0.327 | | **<0.001** | Constant | 11.35 (-0.71 a 23.41) | n/a | 0.064 |
|  | | | | TAPSE | 0.59 (0.26 a 0.91) | 0.505 | **<0.001** |
|  |  |  |  | BMI | -0.44 (-0.73 a -0.15) | -0.421 | **0.004** |
|  |  |  |  | Heart rate | 0.12 (0.02 a 0.22) | 0.321 | **0.020** |
|  | | ^1^ left ventricle global longitudinal strain; ^2^ left atrial reservoir strain; ^3^ left atrial contractile strain; ^4^ right atrial reservoir strain; ^5^ right atrial contractile strain; ^6^ right ventricle free wall longitudinal strain; LVEF: left ventricle ejection fraction; DM: diabetes mellitus; TAPSE: tricuspid annular plane systolic excursion; BP: blood pressure; RA: right atrial; LA: left atrial; LV: left ventricle; BMI: body mass index.  * model´s p  ** not applicable | | | | | |

**SUPPLEMENTARY FILE 6 –** Cardiac function differences in people with epilepsy compared to controls


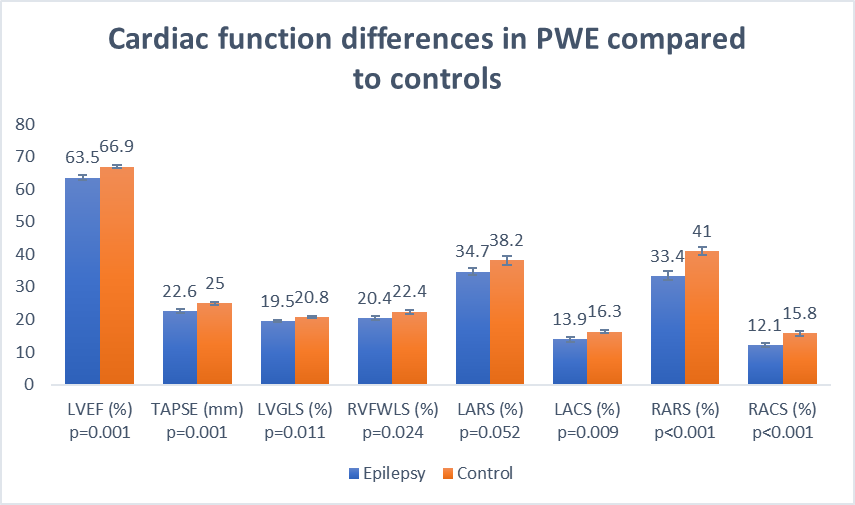


**SUPPLEMENTARY FILE 6**: People with epilepsy, compared to controls, have lower values of LVEF (left ventricular ejection fraction), TAPSE (tricuspid annular plane systolic excursion), LVGLS (left ventricular global longitudinal strain), RVFWLS (right ventricular free wall longitudinal strain), LACS (left atrial contractile strain), RARS (right atrial reservoir strain), and RACS (right atrial contractile strain). LARS (left atrial reservoir strain) was lower in individuals with epilepsy but with a p value of 0.052. TAPSE is expressed in millimeters, LVEF and strain values are expressed as a percentage. All values are represented with standard errors.
